# Evaluation of the effect of piezo‐intracytoplasmic sperm injection on the laboratory, clinical, and neonatal outcomes

**DOI:** 10.1002/rmb2.12324

**Published:** 2020-03-18

**Authors:** Yoshitaka Fujii, Yuji Endo, Shingo Mitsuhata, Momoko Hayashi, Hiroaki Motoyama

**Affiliations:** ^1^ IVF Center Kurashiki Medical Clinic Kurashiki Japan

**Keywords:** assisted reproductive technology, fertilization, ICSI, neonatal outcomes, piezo

## Abstract

**Purpose:**

Limited research has been published on the effect of piezo‐assisted intracytoplasmic sperm injection (P‐ICSI). We evaluated the effect of P‐ICSI on the laboratory, clinical, and neonatal outcomes.

**Methods:**

This retrospective study was based on the data collected between April 2011 and October 2016. Total 1348 mature oocytes from 145 patients were analyzed. Laboratory, clinical, and neonatal outcomes of those given conventional intracytoplasmic sperm injection (C‐ICSI) and those administered P‐ICSI were examined.

**Results:**

P‐ICSI showed significantly more favorable results, with a survival rate of 97.0% (C‐ICSI: 94.1%, *P* < .010) and a fertilization rate of 83.5% (C‐ICSI: 70.6%, *P* < .001). There were no differences in the blastocyst development rate, implantation rate, miscarriage rate, live birth rate, gestational age, birth weight, proportion of male neonates, cesarean section rate, and congenital abnormalities between the two patient groups.

**Conclusions:**

Our comparison of P‐ICSI with C‐ICSI showed that P‐ICSI significantly improved the survival and fertilization.

## Abstract 2

We evaluated the effect of piezo‐assisted intracytoplamic sperm injection (P‐ICSI) on the laboratory, clinical, and neonatal outcomes. Our comparison of P‐ICSI with conventional intracytoplasmic sperm injection (C‐ICSI) showed that P‐ICSI significantly improved the survival and fertilization rates.

## 1 INTRODUCTION

Intracytoplasmic sperm injection (ICSI) is currently used for 60%‐80% of the cycles performed in assisted reproductive technology.^1^, ^2^, ^3^ The high level of standardization achieved in the industry contributes to the extensive use of ICSI.^4^ However, the success rate of ICSI depends on the skills of the technicians. The success of ICSI relies on several processes involved in the micromanipulation technique. Three of the most technically difficult processes are sperm immobilization, penetration of zona pellucida (ZP), and puncture of oocyte membrane (oolemma). During conventional ICSI (C‐ICSI), all these procedures are performed manually. Manual sperm injection has the limitation of poor reproducibility, and there is a need for extensive training to achieve successful results of C‐ICSI.^5^ In addition, the training materials used for ICSI are problematic because there are no animal gametes that are adequate substitutes for a human gamete. Considering this limitation, viable human gametes are ultimately required for training, and this method is ethically questionable. To make C‐ICSI safer and more effective, there is a need for new technology to reduce human involvement.

Piezo‐assisted ICSI (P‐ICSI) is an ICSI procedure that is using an innovative tool. With a very high speed movement of the micropipette created by the piezoelectric elements, P‐ICSI assists with the technically difficult processes of C‐ICSI, such as sperm immobilization, penetration of ZP, and puncture of oolemma. Several authors have reported a significant improvement in the fertilization rate of P‐ICSI as compared to that of C‐ICSI.^6^, ^7^, ^8^ However, limited research has been published on the outcomes of P‐ICSI. This study retrospectively evaluated the laboratory, clinical, and neonatal outcomes of P‐ICSI.

## 2 MATERIALS AND METHODS

We examined two ICSI procedures to determine their clinical efficiency. The two ICSI procedures that were examined were C‐ICSI and P‐ICSI. We compared the survival, fertilization, cleavage, blastocyst at day 5, implantation, pregnancy, miscarriage, live birth, gestational age, birth weight, cesarean section, proportion of male neonates, and congenital abnormalities rates after ICSI.

### 2.1 Patients

This retrospective cohort study included 1348 mature oocytes retrieved from 145 patients with infertility who had been treated at the Kurashiki Medical Clinic between April 2011 and October 2016. Total of 180 treatment cycles were conducted. C‐ICSI was used in 94 treatment cycles conducted for 70 patients between April 2011 and April 2015, and P‐ICSI was used in 86 treatment cycles conducted for 75 patients between April 2015 and October 2016. ICSI was performed in patients with male factor infertility, motile sperm concentration <1 × 10^6^/mL after swim‐up, history of fertilization failure, previous low fertilization rate with conventional in vitro fertilization, or unexplained infertility. Details of these causes are shown in Table 1. Following the recommendation of the Vienna consensus, this study included the following criteria for patient enrollment: age of woman <40 years, ovarian stimulated cycles, use of ejaculated fresh/frozen spermatozoa, and availability of the patient's own fresh oocyte.^9^ This study excluded natural cycles, cycles with poor ICSI fertilization rate (<25%), use of in vitro matured oocyte, thawed/warmed oocyte, giant oocyte, and activated oocyte. To minimize the potential influence of ICSI‐associated technical factors, this study included only those ICSI cycles that were performed by the same two embryologists for C‐ICSI and P‐ICSI.

**Table 1:** Demographic characteristics of the patients

Parameters	Overall	C‐ICSI	P‐ICSI	*P*‐value
Patients	145	70	75	
Causes of infertility^a^				
Male factor (N, % per patients)	108 (74.5)	56 (80.0)	52 (69.3)	NS
Motile sperm concentration <1 × 10^6^/mL after swim‐up (N, % per patients)	47 (32.4)	19 (27.1)	28 (37.3)	NS
History of fertilization failure (N, % per patients)	9 (6.2)	5 (7.1)	4 (5.3)	NS
Previous low fertilization rate with conventional IVF (N, % per patients)	12 (8.3)	8 (11.4)	4 (5.3)	NS
Unexplained infertility (N, % per patients)	39 (26.9)	20 (28.6)	19 (25.3)	NS

Note

No significant difference.

Abbreviations: C‐ICSI, conventional intracytoplasmic sperm injection; NS, not significant; P‐ICSI, piezo‐intracytoplasmic sperm injection.

^a^ Including patients with >1 causes of infertility.

The opt‐out methodology was used for patient enrollment. The Kurashiki Medical Center and Ethics Committees approved the project.

### 2.2 Gamete preparations

Ovarian stimulation was performed using standard gonadotropin‐releasing hormone (GnRH) agonist/follicle‐stimulating hormone (FSH) protocols or a GnRH antagonist/FSH protocol. Vaginal ultrasound‐guided follicle puncture was performed 35‐36 hours after the administration of human chorionic gonadotropin (hCG). The retrieved oocytes were cultured in fertilization medium (Global For Fertilization, LifeGlobal Group). Denudation of the oocytes was performed 3 hours after oocyte retrieval with the use of Cumulase (Origio). The denuded oocytes were transferred to the culture medium (Global, LifeGlobal Group). Only metaphase II oocytes were subjected to injection.

The semen samples were collected by masturbation. Basic semen analysis measured the number of spermatozoa (per unit volume and per ejaculate), motility, and morphology. These are assessed according to World Health Organization (WHO) criteria, updated in 2010.^10^ The semen samples were allowed to liquefy for 30 minutes, and spermatozoa were isolated using centrifugation with Sil‐Select (FertiPro). Then, the motile spermatozoa were recovered by using either the swim‐up or the swim‐side method with ART dish (Nipro). When these methods were impossible, sperm suspension was centrifuged. Prepared sperm samples were deposited in 6% polyvinylpyrrolidone (PVP) (Irvine Scientific) solution.

### 2.3 Conventional ICSI

An inverted microscope (IX‐71; Olympus Corporation) with a micromanipulator (Narishige Inc) was used. The ICSI micropipette (MT‐INJ30; Kitazato Corporation) was connected to a pneumatic injector (IM‐11‐2; Narishige Inc). Small amounts of mineral oil were aspirated into the micropipette by capillary action; then, small amounts of 6% PVP solution were aspirated. Motile sperm were immobilized by crushing the tail between the injection pipette and the dish in 6% PVP solution. An immobilized spermatozoon was slowly aspirated into the injection pipette, tail first. An oocyte was held by holding pipette. With the polar body at either the 12 or 6 o'clock position, the injection pipette was pushed through the ZP far into the oocyte (80% of the oocyte diameter). Small amounts of cytoplasm were aspirated into the micropipette until a sudden flow of cytoplasm into the micropipette occurred. The cytoplasm was injected back into the oocyte with sperm to transfer the sperm into the oocyte. The micropipette was slowly pulled out, ensuring not to release the sperm from the oocyte.

### 2.4 Piezo‐ICSI

An inverted microscope with a micromanipulator equipped with a piezo impact drive unit (MB‐S; Prime Tech Ltd.) was used. The piezo unit was driven by a controller (PMM4G; Prime Tech Ltd.). The operation liquid (Prime Tech Ltd.) was sterilized by filtration using Millex‐LG (Merck Millipore Ltd.), and 1 cm in length was placed in the middle of the injection pipette. The ultrathin walled P‐ICSI micropipette (PINU06‐25FT; Prime Tech Ltd.) was used for injection.^8^ The operation liquid was pushed out to the tip of the micropipette and 6% PVP solution was aspirated, ~200 μm in length, into the micropipette with the aid of a pneumatic injector. Piezo pulses were used to assist sperm immobilization,^6^, ^7^, ^8^, ^11^ penetration of ZP,^6^, ^7^, ^8^, ^11^ and puncture of oolemma.^7^, ^8^, ^11^ Sperm immobilization was performed as follows. In 6% PVP solution, sperm was aspirated into the injection pipette, tail first. Then, sperm was slowly put forward. The pipette tip was pressed against the sperm tail by moving the pipette sideways when the sperm came out of the pipette tip. While pressing the pipette against the sperm tail, continuous piezo pulses were driven to immobilize the sperm. At this time, the pipette was prevented from hitting the sperm head. An oocyte was held by holding pipette. With the polar body at 12 o'clock, the ZP was pierced with continuous piezo pulses. Both, sperm immobilization and ZP penetration were performed using piezo pulses of the same intensity and speed (intensity 2, speed 4). After penetration of ZP, the remnant ZP was expelled, and the sperm was advanced to the tip of the micropipette. The pipette was inserted deep into the oocyte cytoplasm (ooplasm) (80% of the oocyte diameter). To puncture the oolemma, a single piezo pulse was used (intensity 3, speed 1). The sperm was injected into the oocyte with the minimum volume of PVP, and the micropipette was slowly pulled out, ensuring that the sperm was not released from the ooplasm.

### 2.5 Embryo culture

The oocytes treated with C‐ICSI or P‐ICSI were individually placed in droplets of culture medium and covered with mineral oil (Fuso Pharmaceutical Ind.) and cultured at 37°C under 6% CO_2_, 5% O_2_, and 89% N_2_. Fertilization was confirmed on day (D) 1 (17‐19 hours after insemination). Normal fertilization was considered to have occurred when two pronuclei (2PN) and second polar body (2PB) were present. The fertilization rate was calculated as the total number of normally fertilized oocytes per total number of injected oocytes. They were cultured for 3‐6 days in culture medium. Embryo evaluation was performed using the established score guidelines.^12^, ^13^, ^14^ Cleavage was assessed on D2. The blastocyst development rate was assessed on D5. Embryos were placed in EmbryoGlue (Vitrolife Sweden AB) before transfer.

### 2.6 Embryo transfer

Best quality embryos were transferred into the uterus on D3‐6. We performed blastocyst transfer whenever possible. However, cleavage‐stage embryo transfers were performed when it was determined that embryos may develop poorly during extended culture, based on the patient's age, the number, and the morphology of embryos at D3. Surplus good quality embryos were vitrified, as described previously.^15^ Fresh embryo transfer was canceled for patients with signs of ovarian hyperstimulation syndrome or unsuitable endometrium conditions for fresh embryo transfer. The number of embryos to be transferred was basically one. In some cases, two embryos were transferred for women aged ≥35 years. Two embryos were transferred after comprehensively considering multiple factors, such as the patient's age, previous pregnancy history, the morphology of embryos at the time of transfer, and reasonable patient's wishes.

### 2.7 Follow‐up and evaluation index

In order to confirm the establishment of a clinical pregnancy, an ultrasonography check was performed to visualize a gestational sac and fetal heartbeat. Miscarriage was defined as the spontaneous loss of a fetus before the 22nd week of gestation. Live birth rate was defined as live birth delivery cycles divided by the transfer cycles. Preterm birth was defined as birth before the completion of 37 weeks of gestation. Birth weight <2500 g was defined as low birth weight. The duration of pregnancy, mode of delivery, and the weight and sex of the neonate were recorded as the neonatal outcomes.

### 2.8 Statistical analyses

Analyses were performed using the Statcel 2 program (OMS Publishing). Continuous variables are represented as mean ± standard deviation (SD). Frequency and percentage were calculated for the categorical variables, and the between‐group differences were tested using the chi‐squared test and Fisher's exact test when the expected frequencies were ≤5. *P* < .05 was considered statistically significant.

The sample size was based on the primary outcome of the study, the fertilization rate. An analysis of C‐ICSI cycles performed in Kurashiki Medical Clinic prior to this study revealed that the mean number of injected oocytes was 7.8. Sample size was calculated to allow detection of a difference between fertilization rates of 65% and 80% in two groups, with 99.9% power and a two‐tailed 1% significance level. These ICSI fertilization rates were based on the minimum performance (competence) level (≥65%) and aspirational (benchmark) level (≥80%) proposed in the Vienna Consensus.^9^ The required sample size was thus calculated to be 74 per group. Considering that there may be patients with multiple oocyte retrieval cycles during the study period, it was estimated that the number would decrease by 25%; a target of 56 patients per group was therefore determined to be sufficient to compare the two groups. Sample size was calculated using freely available EZR (Easy R).

## 3 RESULTS

Table 2 summarizes the demographic characteristics of the semen. In comparison of characteristics of semen between C‐ICSI and P‐ICSI groups, there were no significant differences in the proportion of patients with oligospermia (45.7% vs 46.7%), asthenospermia (64.3% vs 49.3%), and normospermia (22.9% vs 32.0%). The mean volume (3.2 ± 1.8 vs 2.9 ± 1.6 mL), sperm concentration (50.3 ± 65 ×10^6^/mL vs 37.6 ± 40.1 × 10^6^/mL), and motility (30.3% ± 24.1% vs 35.1% ± 21.7%) of the two groups were comparable. We performed ICSI for patients who were unlikely to expect sufficient fertility with conventional in vitro fertilization, and the rapid forward motility of the C‐ICSI group and P‐ICSI group were as low as 6.2% ± 14.8% and 6.6% ± 11.9%, respectively.

**Table 2:** Demographic characteristics of the semen

Parameters	Overall	C‐ICSI	P‐ICSI	*P*‐value
Patients	145	70	75	
Characteristics of semen^a^				
Oligospermia (N, % per patients)	67 (46.2)	32 (45.7)	35 (46.7)	NS
Asthenospermia (N, % per patients)	82 (56.6)	45 (64.3)	37 (49.3)	NS
Normospermia (N, % per patients)	40 (27.6)	16 (22.9)	24 (32.0)	NS
No. of OPU cycles	180	94	86	
Semen analysis				
Volume (mL, mean ± SD)	3.0 ± 1.7	3.2 ± 1.8	2.9 ± 1.6	NS
Concentration (×10^6^/mL, mean ± SD)	43.4 ± 53.8	50.3 ± 65.5	37.6 ± 40.1	NS
Motility (%, mean ± SD)	32.4 ± 23.2	30.3 ± 24.1	35.1 ± 21.7	NS
Rapid forward motility (%, mean ± SD)	6.3 ± 13.6	6.2 ± 14.8	6.6 ± 11.9%	NS

Note

No significant difference.

Abbreviations: C‐ICSI, conventional intracytoplasmic sperm injection; NS, not significant; P‐ICSI, piezo‐intracytoplasmic sperm injection.

^a^ Including patients with >1 characteristics of semen.

Table 3 summarizes the laboratory and clinical outcomes in the C‐ICSI and P‐ICSI groups. In C‐ICSI, 94 cycles of oocytes retrieval were performed on 70 patients. In P‐ICSI, 86 cycles of oocytes retrieval were performed on 75 patients. The two groups (C‐ICSI and P‐ICSI) were comparable in terms of the average number of oocytes retrieval cycles (1.32 cycles vs 1.15 cycles), the mean age of women at the time of ICSI (35.1 vs 34.7 years), mean number of retrieved oocytes (9.5 vs 9.4), and the mean number of injected oocytes (7.6 vs 7.4). In the C‐ICSI group, 711 oocytes were injected, and 669 oocytes survived after ICSI, with a survival rate of 94.1%. In the P‐ICSI group, 637 oocytes were injected and 618 survived after ICSI, with a survival rate of 97.0%. The survival rate was significantly high in the P‐ICSI group as compared to that in the C‐ICSI group (*P* = .010). The fertilization rate was significantly higher in the P‐ICSI group than in the C‐ICSI group (83.5% vs 70.6%) (*P* = .001). There were no differences between the C‐ICSI and P‐ICSI groups with respect to the cleavage rate (99.0% vs 97.6%), the blastocyst formation at D5 (37.1% vs 42.7%), and the rate of transferred embryos per 2PN oocyte (16.9% vs 13.0%). The rate of D5‐6 embryo transfer cycles per OPU cycles was significantly higher in C‐ICSI than in P‐ICSI groups (66.0% vs 47.7%). The rate of vitrified embryos per fertilized oocyte was significantly lower in C‐ICSI than in P‐ICSI groups (34.9% vs 45.3%). There were no differences between the C‐ICSI and P‐ICSI groups with respect to the implantation (30.6% vs 23.2%), pregnancy (32.1% vs 25.4%), miscarriage (15.4% vs 18.8%), and live birth rates (27.2% vs 20.6%).

**Table 3:** Laboratory and clinical outcomes in the C‐ICSI and P‐ICSI groups

Parameters	Overall	C‐ICSI	P‐ICSI	*P*‐value
Patients	145	70	75	
No. of OPU cycles	180	94	86	
OPU cycles per patients (N, mean ± SD) (range, cycles)	1.23 ± 0.62 (1‐4)	1.32 ± 0.76 (1‐4)	1.15 ± 0.42 (1‐3)	NS
Age of women (years, mean ± SD)	34.9 ± 3.7	35.1 ± 3.5	34.7 ± 4.0	NS
Retrieved oocytes per cycles (N, mean ± SD)	9.5 ± 5.6	9.5 ± 5.7	9.4 ± 5.6	NS
No. of injected oocytes	1348	711	637	
Injected oocytes per cycles (N, mean ± SD)	7.5 ± 4.6	7.6 ± 4.8	7.4 ± 4.4	NS
Oocyte survival rate (%)	1287/1348 (95.5)	669/711 (94.1)	618/637 (97.0)	.010
Fertilization (2PN) rate (%)	1034/1348 (76.7)	502/711 (70.6)	532/637 (83.5)	<.001
Cleavage rate (%)	1016/1034 (98.3)	497/502 (99.0)	519/532 (97.6)	NS
Blastocysts formation at D5 (%)	413/1034 (39.9)	186/502 (37.1)	227/532 (42.7)	NS
No. of transferred embryos/2PN oocytes (%)	154/1034 (14.9)	85/502 (16.9)	69/532 (13.0)	NS
No. of transfer cycles/OPU cycles (%)	144/180 (80)	81/94 (86.2)	63/86 (73.3)	0.030
D3‐4 embryo transfer cycles/OPU cycles (%)	41/180 (22.8)	19/94 (20.2)	22/86 (25.6)	NS
D5‐6 embryo transfer cycles/ OPU cycles (%)	103/180 (57.2)	62/94 (66.0)	41/86 (47.7)	.001
No. of vitrified embryos/2PN oocytes (%)	407/1034 (39.4)	166/502 (34.9)	241/532 (45.3)	<.001
Implantation rate (%)	42/154 (27.3)	26/85 (30.6)	16/69 (23.2)	NS
Pregnancy rate (%)	42/144 (29.2)	26/81 (32.1)	16/63 (25.4)	NS
Miscarriage rate (%)	7/42 (16.7)	4/26 (15.4)	3/16 (18.8)	NS
Live birth rate (%)	35/144 (24.3)	22/81 (27.2)	13/63 (20.6)	NS

Note

Significant at *P* < .05.

Abbreviations: C‐ICSI, conventional intracytoplasmic sperm injection; D, day; NS, not significant; P‐ICSI, piezo‐intracytoplasmic sperm injection; PN, pronucleus.

Table 4 summarizes the outcomes in neonates of the C‐ICSI and P‐ICSI groups born after fresh embryo transfers. Twenty‐two neonates were born in the C‐ICSI group, and 13 were born in the P‐ICSI group. In the P‐ICSI group, information about neonatal outcomes was obtained by 10 neonates. There were no differences between the C‐ICSI and P‐ICSI groups with respect to the mean gestational age (39.4 ± 1.7 vs 39.1 ± 2.1 weeks), preterm birth rate (4.5% vs 10.0%), mean birth weight (2914 ± 388 vs 3102 ± 374 g), low birth weight rate (4.5% vs 10.0%), cesarean section rate (13.4% vs 30.0%), and proportion of male neonates (40.1% vs 30.0%). There were no multiple births or congenital abnormalities in either group.

**Table 4:** Outcomes in neonates of the C‐ICSI and P‐ICSI groups

Delivered neonate data	Over all	C‐ICSI	P‐ICSI	*P*‐value
No. of neonates	35	22	13	‐
No. of neonates for whom information was obtained	32	22	10	‐
Gestational age (weeks; mean ± SD)	39.3 ± 1.8	39.4 ± 1.7	39.1 ± 2.1	NS
Preterm birth rate (%)	2/32 (6.3)	1/22 (4.5)	1/10 (10.0)	NS
Birth weight (g; mean ± SD)	2959 ± 390	2914 ± 388	3102 ± 374	NS
Low birth weight rate (%)	2/32 (6.3)	1/22 (4.5)	1/10 (10.0)	NS
Cesarean section rate (%)	6/32 (18.8)	3/22 (13.4)	3/10 (30.0)	NS
Proportion of male neonates (%)	12/32 (37.5)	9/22 (40.1)	3/10 (30.0)	NS
Multiple birth (%)	0	0	0	‐
Congenital abnormalities (%)	0	0	0	‐

Note

No significant difference.

Abbreviations: C‐ICSI, conventional intracytoplasmic sperm injection; NS, not significant; P‐ICSI, piezo‐intracytoplasmic sperm injection.

## 4 DISCUSSION

Our comparison of P‐ICSI with C‐ICSI showed that P‐ICSI significantly improved the survival and fertilization.

Our clinic started performing ICSI in 1993. We learned from ICSI experiences that C‐ICSI has several challenges that cannot be overcome with operator efforts and techniques. The most important problem was the risk of sudden membrane breakage caused by ZP deformation during ZP passing. When the injection pipette was difficult to penetrate the ZP, the oocyte was extremely deformed. This deformation may increase the internal pressure of the oocyte. After the pipette penetrated the ZP, the oocyte instantaneously returned to the original shape, resulting in the pipette tip being suddenly inserted deep into the oocyte. On certain occasions, the sudden membrane breakage occurs and contributes to the oocyte degeneration. Another was the difficulty in reliably injecting sperm into an oocyte, especially when the oolemma was tough. These problems appeared to need mechanical assistances, leading to the introduction of P‐ICSI.

The survival rate of the P‐ICSI (97.0%) was significantly higher than that of C‐ICSI (94.1%) (Table 3). When the damage rate was calculated from the survival rate of P‐ICSI, it became 3.0%, corresponding to the aspirational benchmark level (≤5%) when evaluated using key performance indicators (KPI) proposed in the Vienna consensus.^9^ Several authors have also reported a significant reduction in the damage rate of P‐ICSI compared with that in C‐ICSI; (P‐ICSI: 11.9%, C‐ICSI: 18.6%),^6^ (P‐ICSI: 1%, C‐ICSI: 10%).^8^ P‐ICSI has unique procedures in terms of the penetration of ZP and puncture of oolemma that can be performed separately. The injection pipette can safely reach the oolemma without sudden membrane breakage caused by ZP deformation during ZP passing because there is no deformation of ZP with P‐ICSI.^6^, ^7^, ^8^, ^11^ Moreover, the breakage of the oolemma is caused by a single piezo pulse, with no need for ooplasma aspiration,^7^, ^11^ and the thin outer diameter of injection pipette results in less physical damage to the oolemma.^8^ In sum, using the piezo‐ICSI techniques may lead to less damage to the oocytes. The risk factors for oocyte degeneration after ICSI may involve the oocyte quality as well as micro tools and individual technical skills. Oocytes with a fragile oolemma often cause oocyte degeneration after ICSI. Within a cohort of retrieved oocytes, the behavior of oolemma during the injection is not uniform. Rosen et al^16^ suggested that not all oocytes are of same quality, and their fate may be largely established before retrieval. They also suggested that very aggressive attempts during ovarian stimulation may result in some oocytes that are already fragile at the time of ICSI, being retrieved from a less‐than‐optimal follicular environment. However, there are few reports on the etiology of fragile oocytes, and it is difficult to control their incidence. We occasionally experienced oocytes that have broken the oolemma before driving the piezo pulse. These oocytes may have oolemma with weak stretching ability. Several authors have reported the reduction in survival rate of oocytes that have non‐stretchable oolemma during P‐ICSI.^17^, ^18^ Two processes may be involved in resealing the hole created in the oolemma during ICSI.^17^ It has been postulated that resealing of the hole in the oolemma may be facilitated by direct contact with outer surfaces of the oolemma, which would take place during post‐ICSI recovery process in fully stretched oolemma.^19^ The resealing occurs quickly. Alternatively, in oocytes, in which oolemma is broken as soon as a pipette is inserted, a large hole may be made in the oolemma and will be resealed by Ca^2+^‐dependent vesicle‐vesicle fusion events, as reported in mouse 3T3 fibroblast cells 20 and sea urchin oocytes.^21^ The resealing occurs slowly. Oocytes degenerate when the harmful extracellular medium (ie high sodium, low potassium, and high calcium with respect to the ooplasm) enters into the ooplasm for a prolonged time through the hole in the oolemma. Therefore, when inserting an injection pipette, it may be possible to improve oocyte viability if we could discover the way to stretch the oolemma as much as possible before breaking. Even the effect of pipette insertion speed on oocyte survival has not been studied to date. Such studies may be possible with P‐ICSI, which is not affected by ZP during pipette insertion.

The normal fertilization rate of P‐ICSI (83.5%) was significantly higher than that of C‐ICSI (70.6%) (Table 3). The fertilization rate of P‐ICSI corresponded to the aspirational benchmark level (≥80%) when evaluated using the KPI proposed in the Vienna consensus.^9^ Several authors have also reported a significant improvement in the fertilization rate of P‐ICSI compared with that of C‐ICSI; (P‐ICSI: 70%, C‐ICSI: 54%),^6^ (P‐ICSI: 90%, C‐ICSI: 83%),^7^ and (P‐ICSI: 89%, C‐ICSI: 68%).^8^ For successful fertilization using ICSI, the two important steps are injection of immobilized spermatozoa and rupture of oolemma.^22^, ^23^, ^24^ With P‐ICSI, immobilization of sperm is accomplished by piezo pulses.^6^ Yanagida et al 25 reported that sperm immobilized with piezo pulses have been shown to stain the sperm head significantly more rapidly with membrane‐impermeable stain than do sperm immobilized by the conventional squeezing method (5.0 vs 42.2 seconds), suggesting that membrane dissolution is more rapid with piezo pulses. Fertilization rate was significantly higher with the piezo method (78.3%) than with the squeezing method (69.7%). They also reported that the Ca^2+^ oscillations in human oocytes due to spermatozoon‐oocyte interaction developed earlier with the piezo method (14.4 minutes) than squeezing method (18.4 minutes). Sperm membrane dissolution appears to be essential for the efflux of oocyte activating factors from the sperm after injection. It seems that the strength of the sperm immobilization method is related to the fertilization rate.^25^ In addition, oolemma can be punctured by applying a single piezo pulse, ensuring the injection of the sperm.^6^ The techniques used with piezo‐ICSI may contribute to successful fertilization.

In our present study, the blastocyst development rate was not different between P‐ICSI and C‐ICSI groups (P‐ICSI: 42.7%, C‐ ICSI: 37.1%) (Table 3). Dumoulin et al reported that blastocyst formation was lower from oocytes that had >6 picoliters of ooplasm aspirated into the injection pipette during the C‐ICSI procedure.^26^ Very recently, Furuhashi et al 27 reported a significant increase in blastocyst development rate with P‐ICSI on D5 and D6 compared to that with C‐ICSI for women >35 years of age (P‐ICSI: 52.4%, C‐ ICSI: 39.6%). They speculated that changes in oolemma viscosity may be related to the women's age. And they suggested that P‐ICSI is effective for patients aged >35 years, as P‐ICSI is less invasive to oocytes of aged women compared with C‐ICSI. In horse, it has been suggested that lower blastocyst quality after C‐ICSI vs P‐ICSI reflects delayed sperm component remodeling and oocyte activation.^28^ However, there are few reports of the effects of the ICSI procedure on blastocyst development and further research is needed.

There were no differences in the implantation rates (C‐ICSI 30.6%, P‐ICSI: 23.2%), the miscarriage rates (C‐ICSI 15.4%, P‐ICSI: 18.8%), and the live birth rates (C‐ICSI 27.2%, P‐ICSI: 20.6%) of the C‐ICSI group and P‐ICSI group (Table 3). Hiraoka and Kitamura 8 reported that the implantation rate of P‐ICSI was significantly higher than that of C‐ICSI (C‐ICSI: 19%, P‐ICSI: 31%). Until the present study, their report is the only report that comparing the implantation rates of C‐ICSI and recent P‐ICSI. In order to evaluate the effect of P‐ICSI on the implantation, miscarriage, and live birth rates, it seems necessary to study more cases in the future.

Information on neonatal outcomes was obtained in 22 newborns in the C‐ICSI group and 10 newborns in the P‐ICSI group (Table 4). There were no differences between the two groups in terms of the gestational age, rate of preterm birth, birth weight, rate of low birth weight, cesarean sections rate, proportion of male neonates, rate of multiple births, and rate of congenital abnormalities. These results should be interpreted with caution owing to the small study size and high risk of bias.

The first report on the use of P‐ICSI that involved mice was published in 1995.^19^ In this procedure, piezo‐activated axial force was applied to the injection pipette that moved forward against the ZP and oolemma to generate minimal damage. However, mercury was usually placed near the tip of the injection pipette to suppress its unwanted lateral movement.^29^ There has been no report that mercury that used in P‐ICSI had a negative effect on fertilization. However, assuming the clinical application of P‐ICSI to humans, mercury is a potential neurotoxin and difficult to handle, store, and manage safely on a clinical field. Modern piezo injectors usually use fluorinert (stable fluorocarbon‐based fluid) instead of mercury.^8^, ^28^, ^30^ These commercialized safe devices and dedicated pipettes for P‐ICSI are now available in Japan. A large number of P‐ICSI has already been performed. Based on the data from the Japanese Society of Clinical Embryologist, 21% (24/121) of Japanese fertility clinics have introduced P‐ICSI in 2016.^31^ We believe that in the future, P‐ICSI will be able to replace C‐ICSI.

In conclusion, P‐ICSI performed in our laboratory was determined to be safe and highly effective for fertilization.

## CONFLICT OF INTEREST

Yoshitaka Fujii, Yuji Endo, Shingo Mitsuhata, Momoko Hayashi, and Hiroaki Motoyama declare that they have no conflict of interest.

## HUMAN RIGHTS STATEMENT AND INFORMED CONSENT

All procedures followed were in accordance with the ethical standards of the responsible committee on human experimentation (institutional and national) and with the Helsinki Declaration of 1964 and its later amendments. Informed consent was obtained from all patients for being included in the study.

## ANIMAL STUDIES

This article does not contain any studies with animal subjects performed by any of the authors.

## APPROVAL BY ETHICS COMMITTEE

The study design was approved by the appropriate ethics committee of Kurashiki Medical Clinic, Okayama, Japan.
